# Mortality, bone density and grip strength: lessons from the past and hope for the future?

**DOI:** 10.1093/rap/rkae046

**Published:** 2024-03-28

**Authors:** Leo D Westbury, Faidra Laskou, Harnish P Patel, Cyrus Cooper, Elaine M Dennison

**Affiliations:** MRC Lifecourse Epidemiology Centre, University of Southampton, Southampton, UK; MRC Lifecourse Epidemiology Centre, University of Southampton, Southampton, UK; NIHR Southampton Biomedical Research Centre, University of Southampton and University Hospital Southampton NHS Foundation Trust, Southampton, UK; MRC Lifecourse Epidemiology Centre, University of Southampton, Southampton, UK; NIHR Southampton Biomedical Research Centre, University of Southampton and University Hospital Southampton NHS Foundation Trust, Southampton, UK; Medicine for Older People, University Hospital Southampton, Southampton, UK; MRC Lifecourse Epidemiology Centre, University of Southampton, Southampton, UK; NIHR Southampton Biomedical Research Centre, University of Southampton and University Hospital Southampton NHS Foundation Trust, Southampton, UK; NIHR Oxford Biomedical Research Centre, University of Oxford, Oxford, UK; MRC Lifecourse Epidemiology Centre, University of Southampton, Southampton, UK; NIHR Southampton Biomedical Research Centre, University of Southampton and University Hospital Southampton NHS Foundation Trust, Southampton, UK; Victoria University of Wellington, Wellington, New Zealand

**Keywords:** mortality, sarcopenia, osteoporosis, epidemiology

## Abstract

**Objectives:**

Therapeutic advances in the management of osteoporosis and sarcopenia have occurred at different rates over the last 2 decades. Here we examine associations between grip strength and BMD with subsequent all-cause and cause-specific mortality in a UK community-dwelling cohort.

**Methods:**

Data from 495 men and 414 women from the Hertfordshire Cohort Study were analysed. Grip strength was assessed by grip dynamometry, femoral neck BMD was ascertained using DXA and deaths were recorded from baseline (1998–2004) until 31 December 2018. Grip strength and BMD in relation to mortality outcomes (all-cause, cardiovascular-related, cancer-related and mortality due to other causes) were examined using Cox regression with adjustment for age and sex.

**Results:**

The mean baseline age of participants was 64.3 years (s.d. 2.5) and 65.9 years (s.d. 2.6) in men and women, respectively. Lower grip strength was associated with increased risk of all-cause mortality [hazard ratio (HR) 1.30 (95% CI 1.06, 1.58), *P* = 0.010] and cardiovascular-related mortality [HR 1.75 (95% CI 1.20, 2.55), *P* = 0.004]. In contrast, BMD was not associated with any of the mortality outcomes (*P* > 0.1 for all associations).

**Conclusion:**

We report strong relationships between grip strength and mortality compared with BMD. We hypothesize that this may reflect better recognition and treatment of low BMD in this cohort.

Key messages Grip strength was related to subsequent mortality in a community-based cohort. In contrast, there was no relationship between baseline BMD and subsequent mortality. We hypothesize that this may reflect better diagnosis and management of osteoporosis.

## Introduction

Musculoskeletal health disorders including osteoporosis and sarcopenia are highly prevalent in older adults [1]. Osteoporosis, a disease characterized by low bone mass and structural deterioration of bone tissue, is the most common chronic metabolic bone disease and is associated with fragility fractures, including fractures at the hip or spine, which have been associated in several studies with excess mortality [2]. Sarcopenia is characterized by progressive and generalized decline in muscle strength, function and muscle mass with increasing age or secondary to disease [3] and is also associated with a range of adverse physical and metabolic outcomes, including excess mortality [4].

Although historically several studies have reported associations between either low BMD or low grip strength and mortality risk, therapeutic advances in the management of osteoporosis and sarcopenia have occurred at different rates over the last 2 decades, with many more therapeutic modalities available for osteoporosis. Recent recognition of a high prevalence of coexistence of both osteoporosis and sarcopenia in individuals has led to the existence of the term ‘osteosarcopenia’, the so-called hazardous duet where adverse consequences are commonly recognized in individuals with the condition [5], with higher mortality risk recognized among individuals with the condition who have sustained a hip fracture [6]. In this study, we examined the relationships between BMD and grip strength and subsequent all-cause and cause-specific mortality in a real-world setting in a UK community-dwelling cohort of participants ages 59–73 years at baseline and followed up for approximately 20 years.

## Methods

### The Hertfordshire Cohort Study (HCS)

The HCS is a study of 2997 women and men born in Hertfordshire (UK) between 1931 and 1939. The participants were all living in Hertfordshire in 1998–2004, when they completed a home interview and clinic visit for a detailed characterization of their health. The HCS received ethical approval from the Hertfordshire and Bedfordshire Local Research Ethics Committee and all participants gave informed consent for the investigations they underwent during the home interview and clinic visit and for researchers to access their medical records in the future [7, 8]. All procedures performed in studies involving human participants were in accordance with the ethical standards of the institutional and/or national research committee and with the 1964 Helsinki Declaration and its later amendments or comparable ethical standards.

### Ascertainment of participant information at the baseline clinic (1998–2004)

At the baseline clinic, grip strength was measured three times on each side using a Jamar hand-held dynamometer (Promedics, Blackburn, UK); the highest of the six measurements was used for analysis. A subgroup of 498 men and 468 women had their BMD assessed at the lumbar spine and proximal femur using a QDR 4500 dual-energy X-ray absorptiometer (Hologic, Marlborough, MA, USA). Current use of bisphosphonates was part of the exclusion criteria for the baseline DXA scan (although ≈15% of women who had a baseline DXA scan were on hormone replacement therapy). Results from the baseline DXA scan were fed back to participants and their general practitioners (GPs) and osteoporosis therapy was recommended if clinically indicated. Among the subgroup who underwent baseline DXA scans, follow-up studies that also involved DXA were conducted in 2011–2012 (*n* = 443) and 2017 (*n* = 224) [8]; at each of these time points, ≈10% of participants reported taking bisphosphonates.

Probable sarcopenia [low grip strength of <27 kg (men), <16 kg (women)] was defined according to the 2019 European Working Group on Sarcopenia in Older People (EWGSOP2) [4]. Osteoporosis was defined according to the World Health Organization criteria as a femoral neck BMD T-score <−2.5. Probable osteosarcopenia was defined as the combination of both low grip strength and osteoporosis.

### Ascertainment of mortality outcomes

The Ethics and Confidentiality Committee of the National Information Governance Board and NHS Digital provided permission to obtain mortality data from HCS participants from baseline until 31 December 2018. Mortality outcomes included all-cause mortality, cancer-related mortality [International Classification of Diseases, Tenth revision (ICD-10) codes for underlying cause: C00–C97], cardiovascular-related mortality (I10–I79) and mortality not due to cancer or cardiovascular causes. (REC reference: 16/EE/0374 01 April 2020 IRAS project ID: 208811).

### Statistical methods

Summary statistics, such as means and s.d.s, medians and interquartile ranges (IQRs) and percentages, were used to describe participant characteristics. Grip strength and femoral neck BMD were normally distributed within each sex; this was confirmed through visual examination of histograms. Cox regression was used to examine grip strength and femoral neck BMD in relation to the following mortality outcomes: all-cause mortality; cardiovascular-related mortality; cancer-related mortality; and mortality not due to cancer or cardiovascular causes. Hazard ratios (HRs), along with their 95% CIs, were derived using these models. Models were adjusted for age and sex; evidence of sex-interaction effects was weak (*P* > 0.2 for all interactions). Analyses were performed using Stata version 17.0 (StataCorp, College Station, TX, USA); *P* < 0.05 was regarded as statistically significant. The sample size was the largest possible, given the data available. The analysis sample comprised participants who underwent the baseline DXA scan (none of these participants were taking bisphosphonates) and were also not on hormone replacement therapy (*n* = 909); all these participants had values for grip strength and femoral neck BMD.

## Results

Table 1 presents the participant characteristics of the analysis sample. The mean age at baseline was 64.3 years (s.d. 2.5) and 65.9 years (s.d. 2.6) for men and women, respectively. The mean grip strength was higher among men compared with women (44.1 *vs* 27.5 kg); this was also the case for femoral neck BMD (0.85 *vs* 0.75 g/cm^2^). The prevalence of EWGSOP2 probable sarcopenia [low grip strength <27 kg (men), <16 kg (women)] was 1% in both men and women, while the prevalence of osteoporosis (femoral neck BMD T-score <−2.5) was 1% in men and 5% in women. No participants had probable osteosarcopenia, defined as the combination of low grip strength and osteoporosis. Approximately 35% of men and 25% of women died during follow-up.

**Table 1.** Participant characteristics of the analysis sample (*N* = 909)

Participant characteristics	Men (*n* = 495)	Women (*n* = 414)
Age, years, mean (s.d.)	64.3 (2.5)	65.9 (2.6)
Grip strength (kg), mean (s.d.)	44.1 (7.3)	27.5 (5.0)
EWGSOP2 probable sarcopenia, %	1	1
Femoral neck BMD, g/cm^2^, mean (s.d.)	0.85 (0.12)	0.75 (0.12)
Osteoporosis, %	1	5
Mortality outcomes, %		
All-cause mortality	35	25
Cancer-related	15	10
Cardiovascular-related	10	5
Other (not cancer or cardiovascular-related)	10	10
Follow-up time, years, median (IQR)	18.8 (16.5–19.3)	17.3 (16.3–17.8)

Percentages for mortality outcomes were rounded to the nearest 5%.

Follow-up time until death or until participants were censored is presented.

EWGSOP2 probable sarcopenia: low grip strength <27 kg (men), <16 kg (women).

Osteoporosis: femoral neck BMD T-score <−2.5.

Age- and sex-adjusted HRs for mortality outcomes per s.d. lower grip strength and femoral neck BMD are presented in Table 2. Lower grip strength was associated with an increased risk of all-cause mortality [HR 1.30 (95% CI 1.06, 1.58), *P* = 0.010] and cardiovascular-related mortality [HR 1.75 (95% CI 1.20, 2.55), *P* = 0.004]. In contrast, femoral neck BMD was not associated with any of the mortality outcomes (*P* > 0.1 for all associations). Mutually adjusted associations were similar when grip strength and femoral neck BMD were included in the same model with age and sex as adjustments (data not shown).

**Table 2.** Risk of mortality outcomes per s.d. lower grip strength and femoral neck BMD (adjusted for age and sex)

Mortality outcome (underlying cause)	Grip strength (z-score)		Femoral neck BMD (z-score)	
	HR (95% CI)	*P*-value	HR (95% CI)	*P*-value
All-cause	1.30 (1.06, 1.58)	0.010	1.05 (0.93, 1.20)	0.422
Cancer	1.01 (0.74, 1.39)	0.932	1.19 (0.97, 1.46)	0.104
Cardiovascular	1.75 (1.20, 2.55)	0.004	1.12 (0.87, 1.44)	0.389
Other (not cancer or cardiovascular)	1.30 (0.92, 1.83)	0.136	0.89 (0.72, 1.10)	0.280

Grip strength and femoral neck BMD were included in separate models; each model was adjusted for age and sex.

## Discussion

In this study we report strong relationships between grip strength and mortality after adjustment for age and sex compared with BMD. We hypothesize that this may reflect better recognition and treatment of low bone density, as evidenced by the proportion of our participants reporting bisphosphonate use in follow-up studies. Our study might be considered to represent a natural experiment, as we fed back DXA results from scans performed at baseline as part of this study to participants and their GPs, representing a form of case-finding. In support of this, we found that among participants who were seen in the clinic in subsequent studies, ≈10% reported current bisphosphonate use. Although we might expect the prevalence of osteoporosis to be slightly higher (25% in women and 6% in men >65 years of age) [9], it is possible that some participants may have been taking a holiday from therapy. Furthermore, we know that drug adherence is typically only 30% at 1 year and lower still in subsequent years. For example, ≈75% of women who used oral bisphosphonates revealed non-adherence within 1 year and 50% discontinued therapy by this time [10]. Our figure of 10% represents participants who have been prescribed bisphosphonates and were currently taking them at the time of our research clinic. At the time of these clinics (2011–2012 and 2017), this was the usual therapy for osteoporosis. Fewer than seven women were on hormone replacement therapy in 2011–2012 (*n* = 433) and in 2017 (*n* = 224) and fewer than six women were on strontium therapy in 2017. Denosumab use was not ascertained in this cohort.

The first full publications on the biological effects of bisphosphonates appeared in 1969 [11], but did not become common in osteoporosis management until the 1990s when etidronate was used, to be followed by more potent bisphosphonates such as alendronate, with the advent of large randomized trials. The management of osteoporosis was facilitated by international consensus regarding a definitional approach by the World Health Organization [12]. In contrast, agreement regarding a definitional approach to sarcopenia has been slower [13] and, to date, fewer therapeutic targets have been identified [14].

It has long been recognized that hip and vertebral osteoporotic fractures are associated with considerable immediate and long-term increased mortality risk, which is associated with immediate complications relating to the fracture and surgical repair in the case of hip fractures, and comorbidity in the case of vertebral fractures [15]. Treatment with bisphosphonates has been associated with a decreased risk of mortality in patients with osteoporotic fractures in some observational studies and in randomized controlled trials of zoledronate therapy following hip fracture, with acute phase response identified as important [16].

We have speculated that relationships between BMD and mortality risk were weaker than between grip strength and mortality risk because there is a recognized diagnosis pathway and commonly used treatment for the condition that might reduce mortality risk. Previous studies have considered whether bisphosphonate use reduces mortality. For example, a recent Taiwanese study, using the Taiwan National Health Insurance Research Database linked to national death registration data in 59 926 patients with osteoporotic vertebral fractures, found that after excluding patients with short-term mortality, patients who had previously received anti-osteoporotic medications had a lower mortality risk [HR 0.84 (95% CI 0.81, 0.88)]. Patients receiving treatment for >3 years had a much lower mortality risk [HR 0.53 (95% CI 0.50–0.57)] regardless of which therapy was used [17]. This followed a previous study using the same database that suggested bisphosphonate use was associated with reduced risk of mortality [18].

Lower grip strength has been associated with higher risk of all-cause mortality in several studies [19]. Furthermore, there is evidence that the combination of low strength and low BMD confers greater risk of adverse health outcomes compared with either condition in isolation. For example, in a study comprising 1044 women, age 75 years at baseline, from the Osteoporosis Prospective Risk Assessment cohort, probable osteosarcopenia (low knee muscle strength <175 Nms and low femoral neck BMD T-score <−1.0) was associated with a higher risk of hip and major osteoporotic fracture and mortality compared with low femoral neck BMD alone [20]. This suggests that additional treatments and interventions to those aimed at addressing low BMD may be required to reduce the risk of adverse health outcomes among individuals with probable osteosarcopenia.

This study has some limitations and strengths. A significant strength of our analysis is the standardized method of assessment of both grip strength and BMD in a large single community–based cohort who have been followed up through linked registries. This ensures almost 100% follow-up. As explained above, our classification of sarcopenia is probable rather than confirmed, as specified according to the EWGSOP2 definition, and this, together with the low prevalence of low muscle strength and bone density in this population, meant that we could not explore the coexistence of both conditions in relation to health outcomes in this study. While participants of the HCS have been shown to be generally representative of the UK population, they are all Caucasian, limiting the generalizability of our results. Regarding therapy for osteoporosis, we do not have information on intermittent annual treatments such as zoledronate. However, our observation that BMD was not associated with mortality over 20 years of follow-up where linked data assure no healthy cohort bias is of interest. Further studies of comparable cohorts are now required.

## Data Availability

Data relating to this study cannot be shared due to consent restrictions.
